# Chronic Diphtheria of the Pharynx

**Published:** 1883-02

**Authors:** 


### Original Translations and Abstracts.

### CHRONIC DIPHTHERIA OF THE PHARYNX.

Notwithstanding the frequency of diphtheritic affections at the present day, we rarely meet with cases where the disease is located in the lower part of the pharynx.

Indeed we rather see that many cases, which do not belong to this category, receive the name diphtheritis in spite of the fact we have long since learned—that all is not diphtheria that " has a whitish look."

Extensive follicular inflammation and superficial catarrhal ulcers, such as occur with the exanthems, are often confounded with diphtheria. On the other hand too little attention is paid to those forms in which the upper parts of the pharyngeal space and that portion covered by the soft palate, as well as the posterior surface of the pharynx itself, are involved in the diphtheritic process, either when the disease is seated solely in this part, leaving the lower portions perfectly free, or where at the beginning of the general propagation only the lower and visible parts are involved.

But when the disease attracts attention by extending to the middle ear, the discovery is made in all probability that the true condition was mistaken for gastric fever, or some rheumatic affection.

The abnormal course which diphtheria usually runs in the upper part of the pharynx has on obvious reason therefor. First, the removal and separation of the diphtheritic membrane are made difficult both on account of the greater friction and contact of the parts and from want of expelling power in the muscles during the act of deglutition; and, secondly, artificial separation by means of gargling, instrumentation, etc., is by no means an easy matter.

Then again the long time which the exudation containing the diphtheritic poison remains upon, and its continual contact with, the tissues keep up the infective process, so that the disease extends to parts that previously were exempt. Just here are the dangers that the disease will become chronic, that repair will not ensue after separation of the membrane and that the inflammation will extend to other localities. The directions in which it extends are many. What is oftenest witnessed in the course of the malady, is blenorrhœa with putrid separation of the membrane, which is then the result of extension from the pharynx into the nose where a process of suppuration ensues, lasting often for weeks.

We see it occur as a sequela of scarlatina, leading quite frequently to other morbid conditions; thus, for instance, the ear, previously free from disease, will become implicated in the process. This condition seldom goes unrecognized, since the large masses of exudation that are thrown off are noticed as much by patient as physician.

But far less often described or observed is a second form of chronic diphtheria—the subject of this article—which I have seen in about twelve cases up to the present date, and of whose precise etiology I have but recently been fully convinced.

Among a large number of cases of pharyngeal ulceration which I treated I found ulcers often of large extent, which were either due to syphilis or had an undoubted tuberculous origin. Several resembled syphilitic ulcers but offered no resemblance to them in point of significance and importance.

Here was seen a rather deep lesion having a mottled yellowish floor, and surrounded by swollen and reddened mucous membrane. Under certain circumstances the ulcer extended rapidly and led to severe constitutional disturbances.

In a small number of cases the lesion was only discoverable by rhinoscopy, while the condition had previously been unrecognized and unsuspected because the lower border of the ulcers was not visible below the soft palate. Only intercurrent disease of the ear or pains in the eye brought the patients under my care. Pains in the eye were common to nearly every case. The patients described them as agonizing and intense, starting from the eyes and darting back over one side of the head.

This pain was present in a marked degree when the ulcers reached the level of the *basis cranii.* The ulcerative process did not usually remain confined to the upper portion of the pharynx, but often extended despite all treatment so as soon to appear below the soft palate, indeed in a few cases to involve the commencement of the œsophagus, so that the whole posterior part of the pharynx from the *basis cranii* to the lower half of the epiglottis was transferred into one immense ulcer. At the same time the lesion had extended laterally to no inconsiderable degree. In these cases the posterior surface of the soft palate was always involved so that certain alterations would be recognized from the side of the mouth, such as redness, sometimes lividity and, on touch, a peculiar brittleness and rigidity of the tissue, especially at the edges, so that the gentlest traction broke it off in strips, thus making the use of Voltolini's palate-knife impossible.

In two of the cases observed by me where fissures occurred near the uvula from tearing the membrane, the wound soon became transformed into an ulcer, leaving a thick cicatrix.

The sufferings of the patients were intense, especially the dysphagia and local pain. Then occurred great depression of the forces and marked emaciation, but without any febrile phenomena. Only in a single case was there fever. Here there were diphtheritic crusts, of recent origin, about an ulcer in the upper part of the pharynx, where the membrane was still exfoliating or could be easily removed. This, however, was the very case that gave me an insight into the whole disease.

There was a remarkable resemblance between these and syphilitic or tubercular ulcers, so much so that some of my colleagues had adopted, in a few cases, an anti-syphilitic or an anti-tuberculous plan of treatment. In one case I myself commenced the former, with, of course, wholly negative results.

The first cases, like the one above described, led me to the correct etiology; and then I was enabled to prove that the patients had either previously suffered from recognized diphtheria, or else a subsequent careful study of the history of the case showed that the existence of diphtheritis was highly probable.

The diphtheritic process upon the tonsils was often, apparently, limited in extent, normal in its course, and non-malignant in character, so that the physician was in no wise prepared for the subsequent morbid developments.

One point was common to all, viz., the ulceration began in the upper part of the pharynx. The explanation of this is by no means difficult, if one pictures to one's self the mode of origin of these ulcers, described at the beginning of this article.

At this point we often meet with disturbances that prevent a normal crisis.

How is it, then, that the disease takes on so destructive a character? The chief *role* in the causation of this is played by the previous constitutional condition, so that we may thus formulate: *should diphtheria attack an individual who is the subject of hereditary syphilis scrofulosis or tuberculosis*, even though the latter be only in its incipiency, *and should the process extend from the upper part of the pharynx, or simultaneously develop, from before backward*, then we find a marked predisposition to the ulcerating form of chronic diphiheritis. This was verified in all of our cases but one. In this one case, that of a married army officer, the history elicited no evidence of a syphilitic taint, nor could an hereditary tendency to tuberculosis be made out.

In this patient, who had suffered from a tonsillar diphtheritis that, apparently, left no evil results, an ulcer developed, whose seat was behind the soft palate on the right side, and which continuously extended downward, appearing under the uvula. It resisted treatment most obstinately. But as the patient was out in all kinds of weather, and had to shout out his commands, I take this to be the reason for the formation of an ulcer.

Concerning the *treatment*, we must distinguish two stages in the affection.

So long as the floor of the ulcer appears fatty, and the adjacent parts are markedly infiltrated, a large amount of exudation co-existing caustics and astringents are positively contraindicated. Powerful antiseptics are to be employed at this period; and the virus that undoubtedly lurks in these ulcers must either be destroyed, or its further influence must be checked. Hence, the secretions and exfoliated masses must be carefully and immediately removed.

The nasal and pharyngeal douche, or spray, can not be used too often.

At times the ulcers must be washed out with sponges or by means of the probang, unless the patient objects to it on the ground that it gives too great pain.

Among the applications is a strong solution of carbolic acid (5 to 8 per cent.) and pulverized iodoforn and boracic acid. Iodoform is of special service; it can be blown into the posterior part of the pharynx through the mouth or nose.

Carbolic acid, in the strength above described, is, of course, only for local application and by means of the probang. Weaker solutions may be applied by means of Trautmann's, Von Troeltsch's, or my own atomizer.

No good results followed the use of solutions of chlorate of potassa.

When diphtheria is present, iodoform is to be unhesitatingly employed. Strong solutions of carbolic acid are not well borne in these cases, but weak solutions are permissable.

As soon as the fatty floor of the ulcer begins to loosen up, and when granulations begin to grow at various points, antiseptics are to be discarded (except as additions to the spray) and caustics are to be employed.

Every part of the ulcer is to be rubbed with the caustic, and the application can at times be carried up into the naso-pharyngeal space, if rhinoscopy is easy of performance. Strong solutions of silver nitrate may be applied by means of the atomizer—Von Troeltsch's preferably.

Galvano-cautery likewise proved of considerable service. This may sometimes be employed very easily in the disease, the course of which is then materially shortened. Cautery with white-hot iron calls forth no reaction. We might expect that the action of the ferment-poison would be annulled by this treatment, and hence, in the earlier stages, its action would be most useful, No difficulty attends this mode of treatment so long as the ulcer lies freely exposed to view.

Antiseptics alone will not serve as a cure of the disease in question.

If syphilis is suspected, postpone your mercury and iodine preparations till later on, for the patient is very weak and anæmic.

The ulcers heal from topical treatment. After their cure, constitutional remedies are in order.—*Dr. Walb, in the Ber. Klin. Woch., Dec.* 11, 1882.

